# Low‐coverage whole‐genome sequencing reveals molecular markers for spawning season and sex identification in Gulf of Maine Atlantic cod (*Gadus morhua*, Linnaeus 1758)

**DOI:** 10.1002/ece3.7878

**Published:** 2021-07-14

**Authors:** Timothy P. O’Donnell, Timothy J. Sullivan

**Affiliations:** ^1^ Gloucester Marine Genomics Institute Gloucester MA USA; ^2^ USDA – National Institute of Food and Agriculture Kansas City MO USA

**Keywords:** Atlantic cod, random forest, sex, SNPs, spawning season

## Abstract

Atlantic cod (*Gadus morhua*, Linnaeus 1758) in the western Gulf of Maine are managed as a single stock despite several lines of evidence supporting two spawning groups (spring and winter) that overlap spatially, while exhibiting seasonal spawning isolation. Low‐coverage whole‐genome sequencing was used to evaluate the genomic population structure of Atlantic cod spawning groups in the western Gulf of Maine and Georges Bank using 222 individuals collected over multiple years. Results indicated low total genomic differentiation, while also showing strong differentiation between spring and winter‐spawning groups at specific regions of the genome. Guided regularized random forest and ranked *F*_ST_ methods were used to select panels of single nucleotide polymorphisms (SNPs) that could reliably distinguish spring and winter‐spawning Atlantic cod (88.5% assignment rate), as well as males and females (95.0% assignment rate) collected in the western Gulf of Maine. These SNP panels represent a valuable tool for fisheries research and management of Atlantic cod in the western Gulf of Maine that will aid investigations of stock production and support accuracy of future assessments.

## Graphical abstract

Atlantic cod in the western Gulf of Maine exist as two spawning groups (spring and winter) that overlap spatially, while exhibiting seasonal spawning isolation. Low‐coverage whole‐genome sequencing was used to identify panels of single nucleotide polymorphisms (SNPs) that could reliably distinguish between spring and winter‐spawning individuals, as well as males and females. These SNP panels represent a valuable tool for fisheries research and management of Atlantic cod in the western Gulf of Maine that will aid investigations of stock production and support accuracy of future assessments.

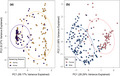

## 1 INTRODUCTION

Fisheries stock assessments and management rely on accurate biological data to effectively estimate population abundances and project the status of stocks. The task of building an accurate stock assessment can become difficult when a species exhibits a complex life history, population structure, or migratory behaviors. Delineating stock boundaries is crucial to unbiased stock assessments (Begg et al., 1999) and becomes even more important when populations exhibit substantial differences in life history. When a stock is delineated, it is treated as one homogenous population; however, that is not the case for species exhibiting subpopulation structure (especially those with differing reproduction and recruitment) and any metapopulation stock assessment operating under an assumption of homogeneity will likely suffer inaccuracies (Dean et al., 2019).

The Atlantic cod (*Gadus morhua*, Linnaeus 1758) is a primary historical example of external forces causing the collapse of a valuable commercial fishery, necessitating swift and effective fisheries management. High fishing pressure in the 1960s and 1970s led to drastic reductions of several Atlantic cod stocks and despite decades of management, many of those stocks are still exhibiting depleted biomass and fishing mortality rates at unsustainable levels (COSEWIC, 2010; NEFSC, 2013). The management of Atlantic cod stocks in the western Gulf of Maine (wGOM) is made difficult because they exist as metapopulations (Dean et al., 2014; Kovach et al., 2010; Zemeckis et al., 2014) but are currently managed as one homogenous unit (NEFSC, 2013), despite evidence that suggests employing substock models would increase productivity yield in the wGOM (Kerr et al., 2010).

A multidisciplinary evaluation of biological stock structure for Atlantic cod in U.S. waters has recently been conducted by the Atlantic Cod Stock Structure Working Group that incorporated data from fishermen's ecological knowledge, life history, genomics, natural markers, and tagging to propose five biological stocks in the U.S.: Georges Bank, southern New England, wGOM and Cape Cod winter spawners, wGOM spring spawners, and the eastern Gulf of Maine (NEFSC, 2021). Each of the proposed stocks is spatially distinct except for the wGOM where there is spatial overlap between spring and winter‐spawning groups that are genetically isolated (Barney et al., 2017; Clucas, Kerr, et al., 2019; Clucas et al., 2019; Kovach et al., 2010) by seasonal differences in spawning behaviors. The spawning groups likely developed due to strong interannual spawning site fidelity (Zemeckis, Hoffman, et al., 2014), group spawning aggregation behaviors (Dean et al., 2014), and were likely facilitated by larval recruitment and/or natal homing. While all Atlantic cod in the wGOM spawn during similar bottom water temperature conditions (6–8℃), “spring” spawning peaks in May‐June and “winter” spawning peaks in November‐December (Dean et al., 2014; Zemeckis, Hoffman, et al., 2014). These seasonal differences in spawning time have large effects on the temperature regimes and oceanic conditions experienced by pelagic larvae and early juveniles, with spring‐spawned Atlantic cod hatching into warm stratified waters and winter‐spawned Atlantic cod hatching into cooling, well‐mixed waters (Huret et al., 2007). It is possible that these conditions could translate to stark differences in production and recruitment success, especially as climate change impacts the wGOM and average summer temperatures continue to climb (Pershing et al., 2015).

The complex population structure and reproductive dynamics observed in the wGOM Atlantic cod have been shown to be perpetuated on a molecular level by selective pressures acting upon chromosomal inversions that affect spawning behavior in the face of gene flow (Barney et al., 2017; Clucas, Kerr, et al., 2019; Clucas, Lou, et al., 2019); however, chromosomal inversions are not unique to Atlantic cod in the wGOM. In Scandinavia, Atlantic cod have repeatedly been characterized by chromosomal inversions linked to migratory behaviors or inshore/offshore ecotypes (Barth et al., 2017; Berg et al., 2016; Karlsen et al., 2013; Kirubakaran et al., 2016; Rodríguez‐Ramilo et al., 2019; Sodeland et al., 2016) and physical water parameters like salinity, oxygen, and temperature (Berg et al., 2015). Similarly, Therkildsen et al. (2013) found that chromosomal inversions in Greenland Atlantic cod corresponded with inshore/offshore ecotypes, as well as temperature and salinity. In Canadian waters, Atlantic cod showed differences in chromosomal inversions associated with migratory behaviors (Sinclair‐Waters, Bradbury, et al., 2018). When examining Atlantic cod collected at several locations throughout its range, chromosomal inversions were associated with ocean basin (Berg et al., 2017; Bradbury et al., 2010, 2013, 2014), migratory behaviors (Hemmer‐Hansen et al., 2013; Kess et al., 2019), or ocean temperature (Bradbury et al., 2010). Chromosomal inversions and selection are common in Atlantic cod, and the genomic differentiation they create may represent a valuable opportunity to develop new tools for Atlantic cod science and management.

Taking advantage of genomic differentiation to create molecular tools for management has been a valuable endeavor for Atlantic cod in other parts of northwest Atlantic. For instance, Sinclair‐Waters, Bentzen, et al. (2018) used SNP genotyping to develop a method that can accurately assign Atlantic cod to a genetically distinct population in a marine protected area (MPA), allowing managers to protect a critical species by designating MPA boundaries in a biologically meaningful way, as well as providing new data to redesign and reevaluate future MPA boundaries. A similar approach could be utilized in the wGOM to develop a molecular tool to identify the spawning season, and potentially sex, of Atlantic cod. A recent nonmolecular tool has been developed to identify the spawning season of wGOM Atlantic cod using the diameter of first annulus of the otolith and a logistic regression model (Dean et al., 2019). This otolith method has provided valuable data to the management of Atlantic cod in the wGOM, including the identification of decreased recruitment of spring‐spawned Atlantic cod over time. Likewise, a molecular tool may augment these results by increasing potential for automation, decreasing processing times, potentially improving accuracy, and adding sex information without sacrificing individuals.

In the present study, we used low‐coverage whole‐genome sequencing to evaluate the genomic population structure of Atlantic cod collected in the wGOM and Georges Bank with samples collected over several years. We report levels of genetic divergence for these cod populations as well as patterns across the genome, showing isolated divergence patterns that are consistent with previous studies. In accordance with a research recommendation from the Atlantic Cod Stock Structure Working Group for the development of rapid assessment tools for assignment of spring and winters spawners in the wGOM (McBride et al., 2021), we then focus on the selection of small numbers of genetic markers to develop powerful assignment tools for Atlantic cod, that can identify spawning group and sex of unknown fish. It is our hope that the results will provide a wealth of new data for understanding Atlantic cod population biology and stock structure. In total, this represents a profound opportunity to add valuable science to support dynamic, biologically meaningful Atlantic cod management in the wGOM.

## 2 MATERIALS AND METHODS

### 2.1 Sample collection

**FIGURE 1:**
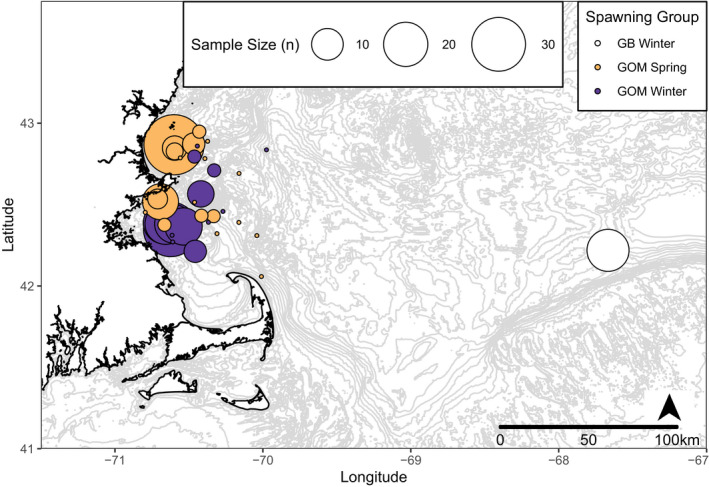
Map of Atlantic cod collection locations with bubble area scaled by the sample size of individual collections (GB =Georges Bank, GOM Spring =western Gulf of Maine spring‐spawning, and GOM Winter =western Gulf of Maine winter spawning)

Caudal fin clips were taken as a source of genetic material from 222 Atlantic cod individuals collected in the wGOM and Georges Bank (Figure 1; Table 1). In the wGOM, Atlantic cod were collected by the Massachusetts Division of Marine Fisheries (MADMF) Industry‐Based Survey via bottom trawl in either the spring (April‐June; *n* = 87) or winter (November‐January; *n* = 117) from 2013–2018 and were stored frozen until laboratory processing. Atlantic cod from Georges Bank (*n* = 18) were collected by commercial fishermen associated with the National Oceanic and Atmospheric Association's Cooperative Research Study Fleet program. All fish chosen for genetic analysis were graded by MADMF biologists as being in spawning condition (i.e., ripe or running) or by commercial fisherman by observing the presence of flowing eggs or milt.

**TABLE 1:** Collection information for Atlantic cod samples used for final genomic analysis with spawning season designated as fish that were reproductively active in spring (April‐June) or winter (November‐January)

Location	Collection Year	Spring	Winter
Georges Bank	2014		18
Gulf of Maine	2013		15
	2014	13	
	2015		36
	2016	45	31
	2017	19	35
	2018	10	
Total		87	135

### 2.2 DNA extraction, library preparation, and sequencing

DNA was extracted from caudal fin tissue using the Macherey‐Nagel genomic DNA NucleoSpin Tissue kit according to manufacturer's instructions and adapted for use on an Eppendorf EPmotion TMX5075 automated liquid handling workstation. Following extraction, all DNA was purified and size‐selected to remove fragments less than ~1,000 bp in length using a paramagnetic bead cleanup with a 0.5:1 bead to sample ratio (KAPA Pure Beads) and a final elution in 10 mM Tris‐HCl, pH 8.0–8.5. The concentration of DNA of each size‐selected sample was estimated using a SpectraMax QuickDrop Micro‐Volume Spectrophotometer (Molecular Devices) prior to library preparation.

A separate 250–450 bp dual‐indexed library was prepared for each individual using the KAPA Hyper Prep Kit adapted for use on the Eppendorf EPmotion TMX5075 automated liquid handling workstation following the manufacturers’ recommendations.

The mode size of each library preparation was estimated using a Fragment Analyzer 5300 (Agilent) and the DNA concentration of each library was estimated using the NEBNext Library Quant Kit for Illumina (New England BioLabs) and run on a QuantStudio12K Flex Real‐Time PCR System (Thermo Fisher). Based on the size and concentration results, libraries were pooled into equimolar concentrations prior to paired end sequencing (2 × 150 bp) in three separate runs using NextSeq 500/550 v2 high output kits on an Illumina NextSeq 500. Libraries were then re‐sequenced using NextSeq 500/550 v2 paired end (2 × 150 bp) mid output kits with pools reassembled based on previous sequence coverage in an attempt to sequence all individuals to between 1x and 4x coverage.

### 2.3 Sequence filtering, mapping, and SNP calling

Bioinformatic processing generally followed the approach outlined by Therkildsen and Palumbi (2017). Forward and reverse raw reads from each lane were concatenated into aggregate files before removing any bases with a quality score threshold below three from the leading and trailing ends using Trimmomatic (Bolger et al., 2014). Additionally, a sliding window approach was used to remove any bases in a four base window that had a mean quality score below 15. Any remaining reads that fell below a minimum length of 36 bases were removed.

Trimmed reads were aligned to the gadMor2 reference genome (Tørresen et al., 2017) using Bowtie2 v2.3.4.3 (Langmead & Salzberg, 2012) and the “very sensitive local” presetting. SAMtools v1.9 (Li et al., 2009) was used to only retain reads with mapping scores greater than 20, sort reads by leftmost coordinates, and merge paired and unpaired reads. Read ends were soft clipped to retain only the read with the highest quality score in overlapping regions using the clipOverlap program in BAMUTIL v1.0.14 (Jun et al., 2015) to avoid double counting sequence support during SNP calling. Duplicate reads (reads that originated from a single fragment of DNA) were removed using the MarkDuplicates module of PICARD Tools v2.20.4 (Broad Institute, 2019). The coverage and mapping depth for each individual was estimated using the SAMtools mpileup module (Li et al., 2009) with the options of counting orphans, disabling probabilistic realignment of base alignment quality, and a minimum base quality of 0. The mpileup results were processed with a custom script to estimate mapping depth after excluding positions with >4× the mean mapping depth, which likely represented a repetitive sequence. When all individuals had been sequenced to ~1× depth, PICARD Tools were used to add read groups and create index files for each individual.

Reads for each individual were aggregated and realigned around indels using IndelRealigner within GATK v3.8 (McKenna et al., 2010) prior to calling SNPs at sites with a probability of <1^–6^ being monomorphic based on the mapped reads for all 222 individuals using ANGSD v0.930 (Korneliussen et al., 2014). The following filters and parameters were used in the ANGSD analysis: Sites with a total read depth <50 and >500 were excluded, and the retained sites were further filtered by setting a minimum number of 150 individuals with data at each site and a minimum quality score of 20. Individual genotype posterior probabilities and genotype likelihoods in Beagle format were generated only for loci with a minor global allele frequency of ≥5%. Additionally, major and minor alleles were inferred from genotype likelihoods across all individuals at all SNPs and any SNP loci that were out of Hardy‐Weinberg Equilibrium (HWE) at *p* ≤ 1^–6^ were removed. Genotype likelihood estimation and SNP calling were repeated in the same fashion for individuals collected in the wGOM (excluding Georges Bank fish).

### 2.4 Population genomics

To produce a visualization of potential Atlantic cod differentiation, a covariance matrix between all individuals was produced based on genotype likelihoods from ANGSD in PCAngsd (Meisner & Albrechtsen, 2018) and resolved into principal components using the *eigen* function in R 4.0.3 (R Core Team, 2020). The resulting principal component analysis (PCA) was plotted along the first two principal components in R using *ggplot2* (Wickham, 2016). The PCA was performed with all individuals included and also with only wGOM individuals (removing those from Georges Bank). Individuals in the PCA were colored according to their spawning season (and location when Georges Bank was included) but also by sex to examine any genome‐wide differentiation according to spawning season or sex. PCAngsd was also used to detect SNP loci under selection using the pcadapt model with p‐values from resulting chi‐squared tests plotted as the negative log of p across all linkage groups (LGs) in R using *ggplot2*. A significance threshold of *p* ≤ 1.63 × 10^–8^ was set using a Bonferroni correction for the number of SNP loci and α = 0.05.

To examine genetic differentiation among Atlantic cod globally and at specific regions across the entire genome, locus‐by‐locus pairwise *F*_ST_ estimates were generated in ANGSD based on genotype likelihood data. Analyses were run with all individuals separated by Georges Bank, spring‐spawning, and winter‐spawning individuals, but also with only wGOM individuals (excluding Georges Bank fish) grouped by spawning season (spring versus winter) and sex (male versus female). Genotype likelihoods were estimated in ANGSD as previously described but for cod individuals separated into the aforementioned groups (stock, spawning season, sex) and used to compute posterior probabilities of sample allele frequency for each group. Then, the allele frequency likelihoods were used to generate a folded site frequency spectrum using the gadMor2 reference genome (Tørresen et al., 2017) as the ancestral state and two‐dimensional site frequency spectra were computed between all pairwise comparisons. *F*_ST_ estimates at each locus between all pairwise comparisons were generated from the two‐dimensional site frequency spectra in ANGSD and global weighted *F*_ST_ estimated were calculated in a custom R script while per‐site estimates of *F*_ST_ were summarized in a sliding window analysis in ANGSD using a window size of 1,000 and a step size of 100 for visualization. The sliding window output for wGOM individuals comparing spawning season and sex was plotted across all LGs using the *ggplot2* package in R.

### 2.5 Informative loci selection

Two methods were used to select informative SNP loci for distinguishing between male and female, as well as spring and winter‐spawning Atlantic cod in the wGOM: ranked *F*_ST_ values and Guided Regularized Random Forest (GRRF). For the ranked *F*_ST_ method, pairwise *F*_ST_ values at each locus for spawning season and sex comparisons were ranked from highest to lowest, assuming that the highest ranked loci would provide the most power to differentiate the groups. The second approach, GRRF (Deng & Runger, 2013), used random forest, which is an ensemble learning technique that utilizes many series of decision trees for classification and can assign importance scores to a set of features (loci). The GRRF technique uses importance scores from a previous random forest run to guide the regularized random forest for loci selection and ranking. To begin loci evaluation with GRRF, only loci with a positive pairwise *F*_ST_ value were considered and all work was conducted within the *RRF* package (Deng, 2013) in R. Missing data were imputed using random forest with the response variable set to sex or spawning season and using ten iterations with 2,000 trees consistent with Sylvester et al. (2018). Random forest was run using 5,000 trees and the number of variables randomly sampled as candidates at each split equal to the square root of the number of loci evaluated. The GRRF was run ten separate times with 5,000 trees, and a coefficient of regulation for each locus dictated by importance scores from the random forest run and gamma=0.3. After each GRRF run, any locus with a positive mean decrease accuracy (importance score) was considered a selected locus. The final list of selected loci were any loci with positive importance scores from any of the ten GRRF runs; selected loci were aggregated and ranked according to mean importance scores across all runs.

### 2.6 Evaluating population assignment accuracy

The total number of loci selected from the GRRF runs was used to compare to the ranked *F*_ST_ method so an equal number of top‐ranked *F*_ST_ loci were also selected for evaluation. The genotypes of selected loci by ranked *F*_ST_ and GRRF were pulled out of the total data set, and any missing data were again imputed using random forest in the method described above. Assignment accuracy for each selection method was estimated using K‐fold cross‐validation and implemented in the R package *assignPOP* (Chen et al., 2018). In K‐fold cross‐validation, all individuals are randomly divided into K groups where one group serves as the test individuals and the remaining groups are used as training individuals to build the predictive model. This method ensures that test and training individuals are independent and every individual is guaranteed to be tested once, avoiding upward biases of population assignment often associated with other tests where training and test data sets are nonindependent (Anderson, 2010). The K‐fold cross‐validations were run so that K = 2–10 for each set of selected loci tested with loci tested five at a time starting with the top five ranked loci (by *F*_ST_ or GRRF), proceeding to the top ten ranked loci, and so on until in increments of five until the total number of selected loci was evaluated. The mean assignment rate was calculated across all values of K for each set of loci tested and plotted using *ggplot2* in R.

### 2.7 Reevaluating sex and spawning season differences using selected loci

After identifying the locus suites that were most accurate and practical for assigning wGOM Atlantic cod to a sex or spawning season, genotype likelihood data for only those locus suites were used to produce separate sex and spawning season covariance matrices in PCAngsd and further principal components using the *prcomp* function in R. The results were plotted across two principal components with normal data ellipses around each group using *ggbiplot* (Vu, 2011) in R.

## 3 RESULTS

### 3.1 Genomic sequencing data quality

The 222 individuals in the Atlantic cod dataset received a mean of 3.5 Gbp and 22.3 million reads. On average, 4.5% of bases were removed from the raw reads following quality trimming and 94.3% of the reads mapped to the gadMor2 reference genome. The average coverage of the reference genome across all individuals was 1.2× (range: 0.5–3.6), which yielded 3,760,540 called SNPs after 21 loci were removed due to failure to meet HWE expectations.

### 3.2 Genomic differentiation

**FIGURE 2:**
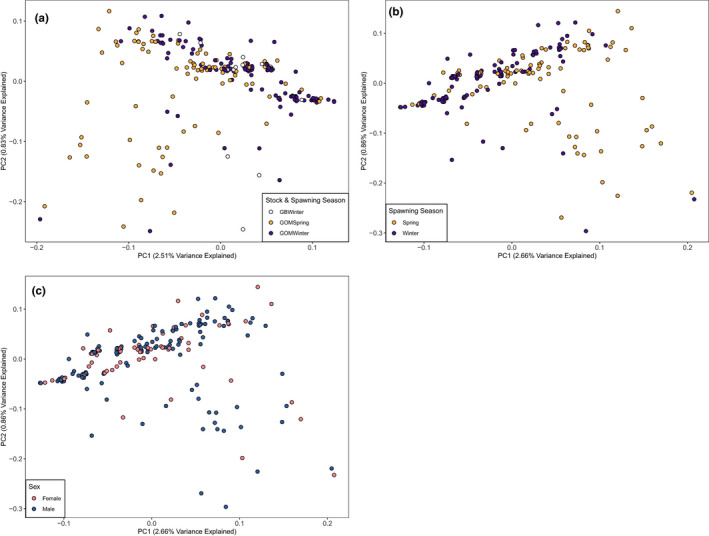
Principal component analysis with (a) all Atlantic cod individuals included and data points colored according to collection location and spawning season or principal component analysis for (b) only western Gulf of Maine individuals with data points colored according to spawning season and (c) sex

Pairwise comparisons of *F*_ST_ between spring and winter‐spawning Atlantic cod as well as Georges Bank fish were low, indicating little genetic differentiation among the groups (Table 2; *F*_ST_: 0.003–0.008) with the largest genetic differentiation occurring between wGOM spring‐spawning Atlantic cod and Atlantic cod collected from Georges Bank. Plotting individuals across the first two PCA axes showed some clustering of points when all individuals were used (Figure 2a) and when only examining Atlantic cod collected in the wGOM (Figure 2b); however, the observed clusters were not directly associated with spawning season, collection location, or sex (Figure 2c), and the variation explained by the two axes was minimal (~3.5%).

**TABLE 2:** Pairwise *F*_ST_ values for Atlantic cod when collections are combined over all collection years, wGOM spring =western Gulf of Maine spring‐spawning and wGOM winter =western Gulf of Maine winter spawning

	wGOM winter	wGOM spring
wGOM spring	0.006	
Georges Bank	0.003	0.008

**FIGURE 3:**
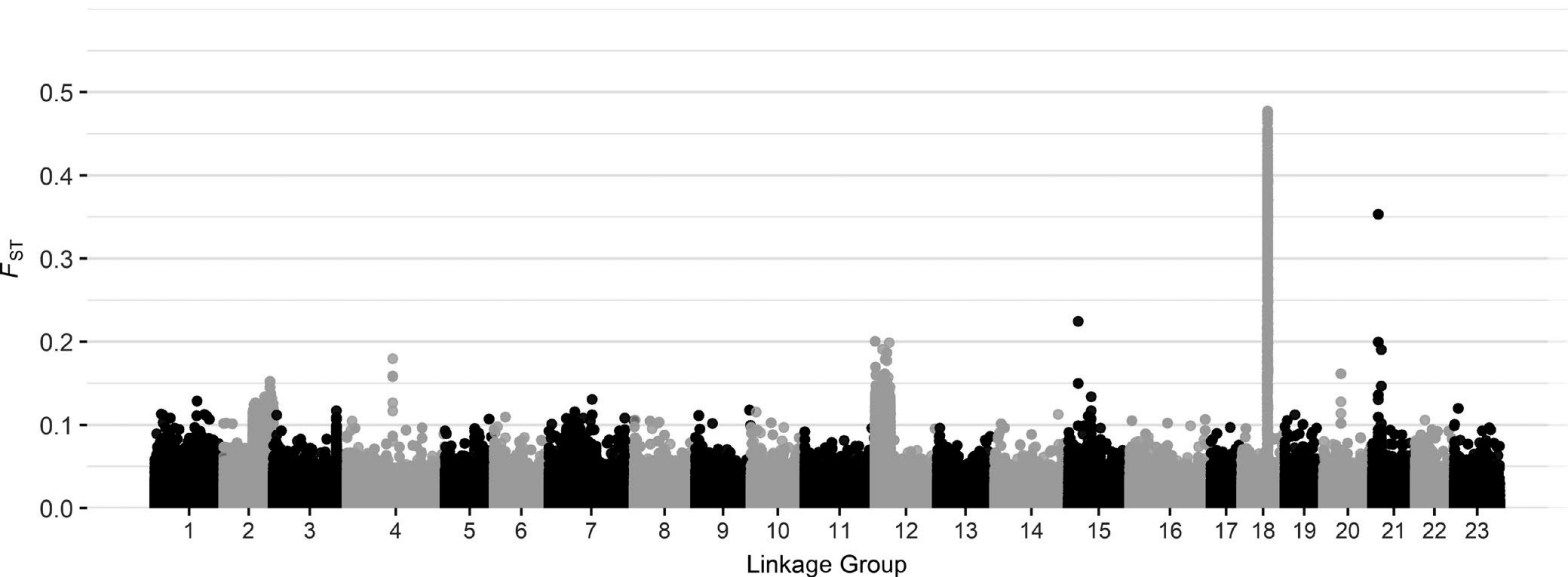
Genome‐wide association with locus‐by‐locus *F*_ST_ values across all 23 linkage groups for comparisons between spring and winter‐spawning Atlantic cod collected in the western Gulf of Maine

**FIGURE 4:**
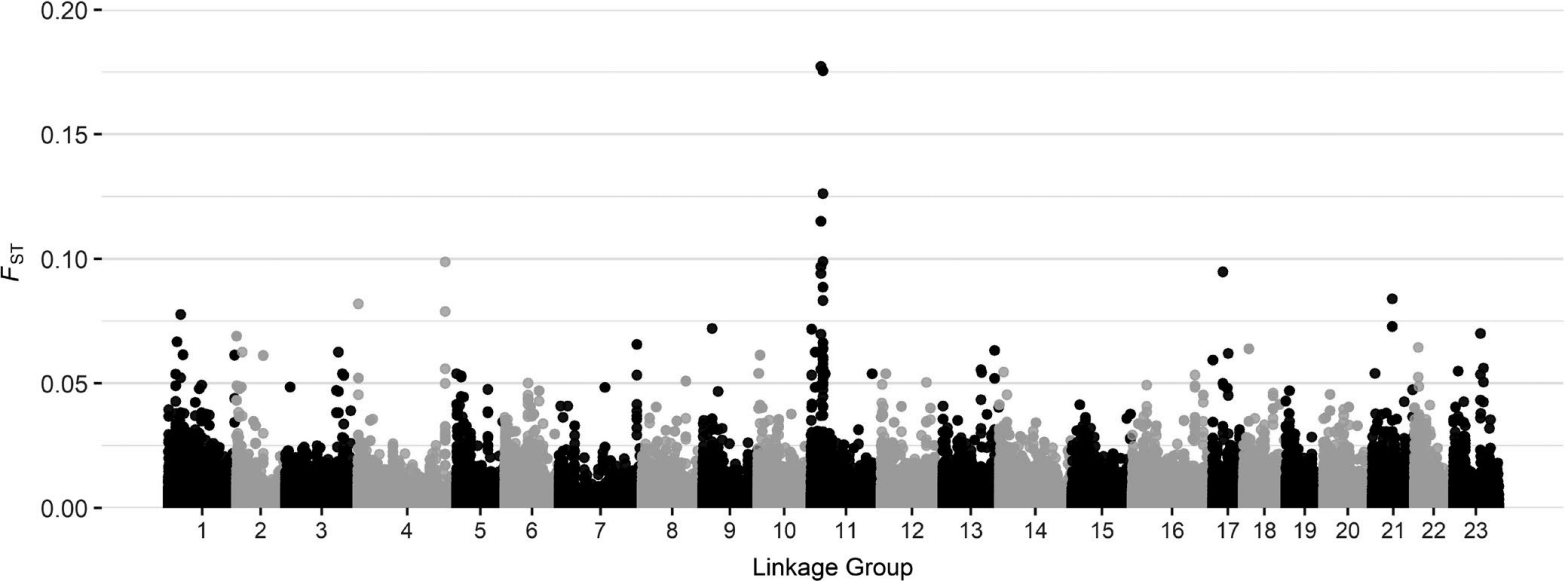
Genome‐wide association with locus‐by‐locus *F*_ST_ values across all 23 linkage groups for comparisons between male and female Atlantic cod collected in the western Gulf of Maine

**FIGURE 5:**
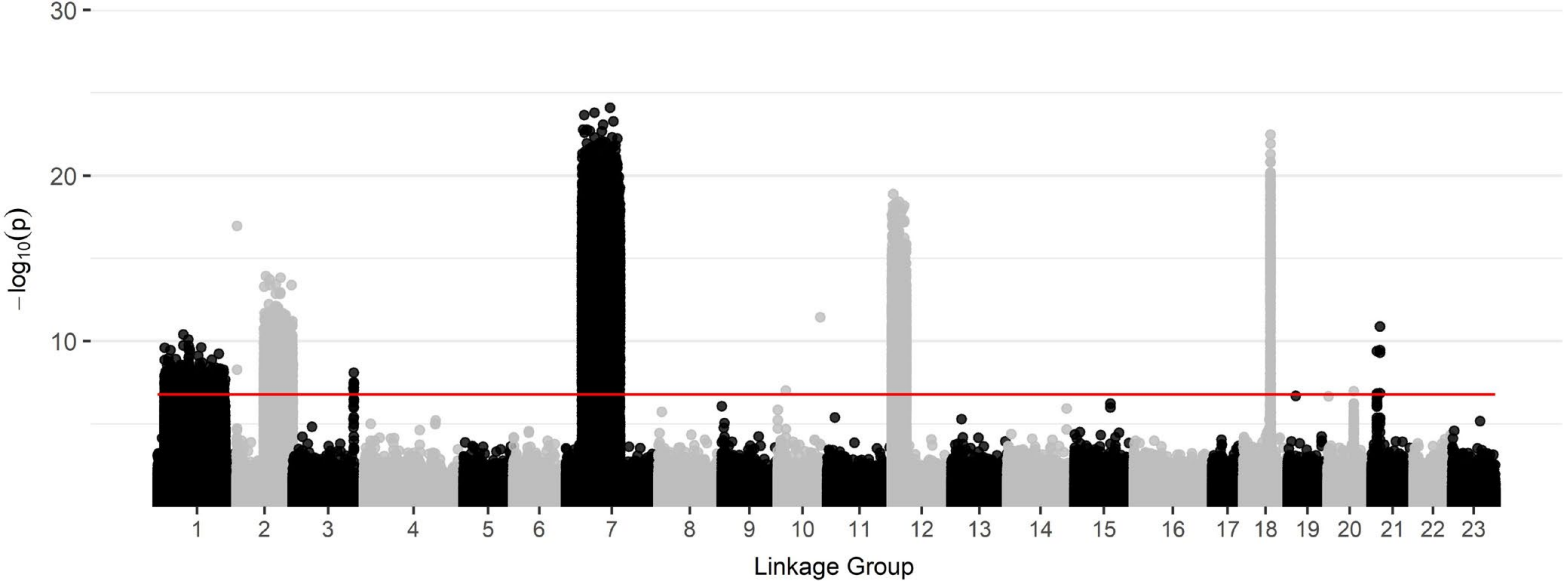
Selection scan results displaying the ‐log_10_(p) for each SNP loci based on a chi‐squared test across all 23 linkage groups for all Atlantic cod collected in the western Gulf of Maine with a red significance line denoting *p* ≤ 1.63 × 10^–8^ set using a Bonferroni correction for the number of SNP loci and α = 0.05

While the genetic distance between spring and winter‐spawning Atlantic cod in the wGOM was negligible on a genome‐wide scale, estimating locus‐by‐locus *F*_ST_ values between spring and winter fish showed that there were isolated areas of the genome (LGs 2, 12, 21, and most strongly 18) showing elevated levels of differentiation above the general baseline (Figure 3). Examining differentiation across the genome for male and female Atlantic cod captured in the wGOM revealed a peak of elevated genetic distance on LG 11 (Figure 4); however, the baseline and the peak *F*_ST_ values between males and females were much lower relative to those covered during the seasonal comparison. There were several areas of the genome under significant selection likely associated with chromosomal inversions or selection on LGs 1, 2, 3, 7, 12, 18, and 21 (Figure 5), many of which overlap with the high areas of differentiation observed between spring and winter‐spawned Atlantic cod.

### 3.3 Marker selection, validation, and individual assignment

**FIGURE 6:**
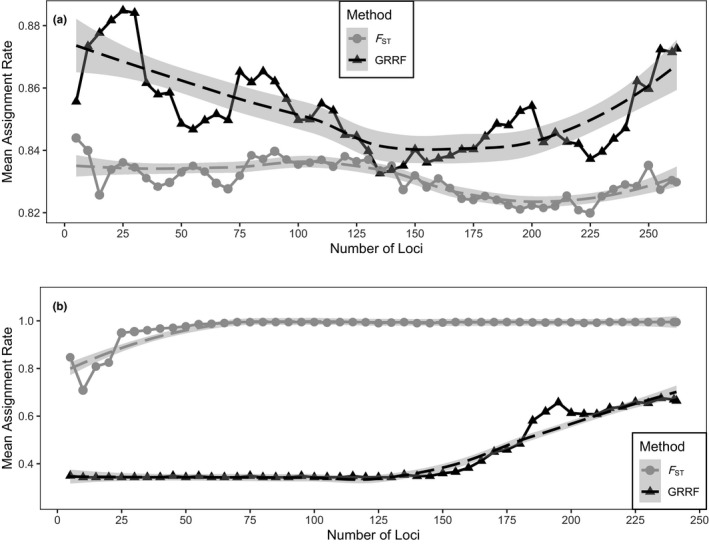
Mean assignment rate for all locus sets selected by Guided Regularized Random Forest and ranked *F*_ST_ methods to differentiate (a) spring and winter‐spawning Atlantic cod and (b) male and female Atlantic cod in the western Gulf of Maine

**FIGURE 7:**
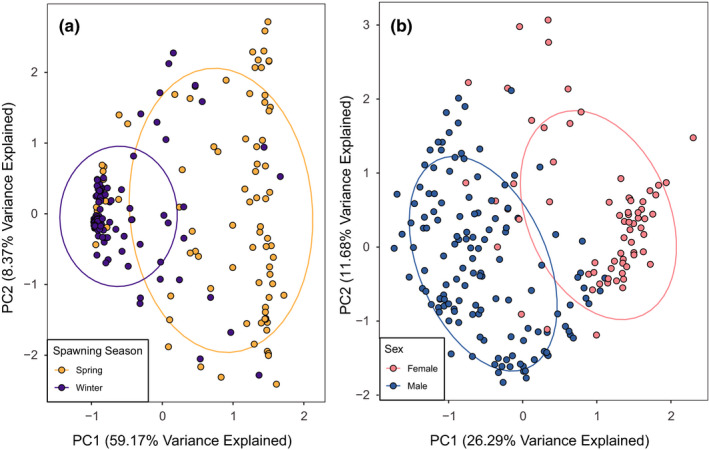
Principal component analysis of western Gulf of Maine Atlantic cod individuals using selected loci to differentiate between (a) spawning season and (b) sex

The GRRF method identified a total of 262 SNP loci with positive importance scores among the ten individual runs that suggested they could distinguish spring and winter‐spawning Atlantic cod in the wGOM with 27 (10.3%) being loci under selection. All 23 LGs were represented in the 262 loci with the majority of the SNPs being located on LG18 (*n* = 50; 19.1%). Mean assignment rate was maximized at 88.5% (Figure 6a) when using the 25 most important loci (6 under selection; 24.0%) according to GRRF, which generally outperformed the ranked *F*_ST_ method for assigning Atlantic cod to spring or winter‐spawning seasons. The population assignment rate using the ranked *F*_ST_ method ranged from 82.0%–84.4% for each set of loci tested. Assignment rate trends for the GRRF method showed a greater fluctuation across the number of loci evaluated relative to the ranked *F*_ST_ method and generally decreased following the peak at 25 loci until ~150 before showing an increase as the number of loci increased from ~150–262. There were only two points (loci = 135 and 140) when the ranked *F*_ST_ method outperformed the GRRF method for population assignment between spring and winter‐spawning Atlantic cod. The 25 loci selected by GRRF that achieved a mean assignment rate of 88.5% between spring and winter‐spawning Atlantic cod (Table 3) represented nine different LGs across the genome (56.0% from LG18) and was much more effective in partitioning spring and winter‐spawning Atlantic cod relative to using all SNP loci. The increase in power to differentiate spawning season in wGOM Atlantic cod was evident when the PCA was reanalyzed using only the suite of 25 loci (Figure 7a). There was little overlap in the normal ellipses when separated by spring and winter spawners and the first two principal components explained 67.5% of the variance.

**TABLE 3:** List of SNP loci used for assigning GOM Atlantic cod to spawning season (i.e., spring or winter‐spawning groups) and sex (males or females) with loci naming conventions of LG followed by the position on each LG and loci listed in order of importance scores

Spawning Season		Sex	
Locus	LG	Locus	LG
LG18_17175832	LG18	LG11_11886873	LG11
LG02_21785361	LG02	LG11_11895561	LG11
LG18_17157703	LG18	LG11_11884313	LG11
LG18_17158133	LG18	LG11_11885753	LG11
LG05_17846762	LG05	LG11_11897588	LG11
LG15_9492450	LG15	LG12_7037474	LG12
LG07_23504388	LG07	LG23_10281376	LG23
LG04_26367477	LG04	LG15_22544499	LG15
LG12_20137107	LG12	LG08_24591305	LG08
LG18_17085587	LG18	LG04_27006645	LG04
LG18_17085503	LG18	LG18_5572934	LG18
LG18_11596188	LG18	LG20_22855211	LG20
LG18_17162871	LG18	LG20_15177680	LG20
LG04_30679825	LG04	LG13_6650832	LG13
LG18_17161121	LG18	LG05_10106110	LG05
LG18_17175640	LG18	LG11_11897513	LG11
LG01_12231215	LG01	LG01_6580190	LG01
LG18_17169811	LG18	LG11_4369856	LG11
LG02_17306759	LG02	LG17_4710806	LG17
LG15_18095655	LG15	LG11_11884389	LG11
LG18_17083513	LG18	LG23_9860028	LG23
LG18_17167765	LG18	LG12_22022616	LG12
LG14_18589849	LG14	LG03_13055640	LG03
LG18_17100075	LG18	LG11_11897519	LG11
LG18_17078527	LG18	LG21_7884830	LG21

When identifying informative loci for distinguishing between sexes of wGOM Atlantic cod, the ranked *F*_ST_ method outperformed GRRF. The GRRF method identified a total of 241 loci (15 under selection; 6.2%) across the ten runs that spanned all LGs. When using the ranked *F*_ST_ method, the top‐ranked 241 loci consistently outperformed the GRRF method for assigning sex to Atlantic cod with an assignment rate of 70.9% to 99.5% across all loci tested. The assignment rate increased to 95.0% by 25 loci and only marginally increased despite the inclusion of additional loci (Figure 6b). When using GRRF, the assignment rate was constant at ~34% until it began to steadily increase to a level of 67% between 150 and 241 loci. Given that the ranked *F*_ST_ method consistently outperformed GRRF when assigning a sex to Atlantic cod and the negligible change in assignment rate when more loci were added beyond 25, the top‐ranked 25 loci by *F*_ST_ (0 under selection) were used as a suite to differentiate males and females (Table 3) and represented 14 different LGs with the majority of SNPs (36.0%) located on LG11. The power of the top 25 loci to differentiate sex was clear when conducting a PCA using only the SNPs selected by *F*_ST_, showing little overlap between males and females and explaining 38.0% of the variation (Figure 7b).

## 4 DISCUSSION

When considering Atlantic cod stock structure among the wGOM and Georges Bank, overall genomic differentiation was low with *F*_ST_ values being similar to, albeit slightly lower than, the neutral *F*_ST_ values calculated by Clucas, Lou, et al. (2019) using a similar sequencing approach. While the level of genomic differentiation between the studies is similar, the lower values observed in the present evaluation could have been influenced by a larger sample size in the wGOM Atlantic cod spawning groups that were collected over several time points, dampening potential differentiation that can occur from sampling in a single time‐point.

The low genetic differentiation observed here also coincides with observed levels of neutral variation in previous studies (Barney et al., 2017; Clucas, Kerr, et al., 2019; Clucas, Lou, et al., 2019; Kovach et al., 2010) and indicates that there is potential gene flow occurring between the seasonal spawning groups in the wGOM. While limited adult movement and strong spawning site fidelity promote stock structure and metapopulations for Atlantic cod (Zemeckis, Hoffman, et al., 2014), there are likely a small number of “migrants” among the seasonal spawning groups in the wGOM that exhibit spawning behavior in the opposite season of their natal spawning season, which decreases genetic distance between spring and winter‐spawned cod. While there is evidence of gene flow between groups of Atlantic cod collected in the northwest Atlantic, neutral genetic variation is only one piece of evidence for the delineation of fish stocks, which should ideally be a multidisciplinary approach (Begg & Waldman, 1999; Zemeckis et al., 2014), that includes differences in life history, natural markers, tagging, and fishermen knowledge, all of which support separating Atlantic cod in the western wGOM into spring and winter‐spawning stocks with Georges Bank Atlantic cod as a separate stock (NEFSC, 2021).

Some of the strongest evidence to support the delineation of spring and winter‐spawning Atlantic cod in the wGOM is presented here and in previous studies (Clucas, Kerr, et al., 2019; Clucas, Lou, et al., 2019; Kovach et al., 2010) in the form of strong genetic differentiation at specific locations throughout the genome that are associated with chromosomal inversions and selection. The location of peaks in genetic differentiation observed in the present study between spring and winter‐spawning Atlantic cod generally coincided with those observed by Clucas, Lou, et al. (2019). The largest contrast between results observed by Clucas, Lou, et al. (2019) and the current evaluation occurs on LGs 7 and 12. While significant selection was detected in both LGs, a peak in genetic differentiation was only observed on LG12 in the current evaluation and none on LG 7, whereas Clucas, Lou, et al. (2019) observed a peak on LG 7 and not on LG 12. As previously discussed, the observed selection in Atlantic cod in LGs 1, 2, 7, and 12 is likely driven by documented chromosomal inversions that have persisted in response to water chemistry parameters including temperature, salinity, and oxygen (Berg et al., 2015; Bradbury et al., 2010; Therkildsen et al., 2013). It is unclear whether SNPs under selection on LGs 3, 18, and 21 represent chromosomal inversions or just areas of strong selection. Many of the genes found on these regions are crucial to the production or reception of reproductive hormones and selective pressures causing differentiation at these loci are likely the cause of the prezygotic isolation between spring and winter‐spawning Atlantic cod in the wGOM (Clucas, Lou, et al., 2019).

The GRRF method routinely outperformed ranked *F*_ST_ when assigning to Atlantic cod to spawning season, which is similar to past findings when differentiating population segments or phenotypes in fish populations (Brieuc et al., 2015; Sinclair‐Waters, Bentzen, et al., 2018; Sylvester et al., 2018). During the present evaluation for Atlantic cod spawning season, the GRRF method selected SNP loci across a wider spread of the genome relative to the ranked *F*_ST_ method, which was limited to two LGs. When using a ranked *F*_ST_ method, Sinclair‐Waters, Bentzen, et al. (2018) saw improved assignment rate of Atlantic cod to a unique population segment in an MPA when SNP loci were filtered for linkage disequilibrium, which likely made more varied loci available for evaluation; however, in that case, GRRF still outperformed both ranked *F*_ST_‐based methods whether or not they were filtered for linkage disequilibrium.

When assigning wGOM Atlantic cod to males and female, the results were the opposite relative to spawning season designation with the ranked *F*_ST_ method producing a locus suite with the greatest assignment rate (95.0%). When considering the final suite of 25 SNPs selected using the ranked *F*_ST_ method for identifying sex, 8 SNPs were located in a previously documented 55kb region on LG 11 that contained genes segregating according to an XX‐XY system in Atlantic cod (Star et al., 2016). Additionally, 4 of the SNPs matched loci previously identified by Star et al. (2016) in a similar set of sex discriminating loci for Atlantic cod collected in Norway and Iceland. Independent evaluations finding the same important region in the genome for sex discrimination are confirmation that genetic sex determination is consistent among ocean basins and is an ancestral trait that was retained despite subsequent population fragmentation. In fact, Kirubakaran et al. (2019) described a true male‐specific 9 kb region consisting of one gene on LG 11 that is flanked by the SNPs used in the sex‐determining suite here and estimated that it evolved in gadoids ~45 million years ago. Using the GRRF method was ineffective at discriminating between sexes in Atlantic cod and the contrasting performance of the GRRF and ranked *F*_ST_ methods for selecting informative SNP loci depending on trait (spawning season or sex) warrants further consideration.

The drastic change in relative performance of the GRRF and ranked *F*_ST_ methods for finding informative loci between the two comparisons could be explained by a number of factors. The most obvious difference between the scenarios was the level and landscape of differentiation across the genome when comparing different sexes and different spawning seasons. The large peak on LG 18 between spawning groups narrowed the field of available loci when using the ranked *F*_ST_ method, and the generally low level of differentiation among sexes may have made it difficult for meaningful trees to take root in the GRRF method for distinguishing males and females.

Another factor that may play a part in the efficiency of the GRRF method is the complexity of the trait being examined. The random forest technique has traditionally been used to distinguish between phenotypes of complex polygenic traits (Bureau et al., 2003). As established here and by Clucas, Lou, et al. (2019), spawning season determination in Atlantic cod is a complex process that involves several genes dispersed throughout the genome. Conversely, sex determination in Atlantic cod is controlled by a single gene (Kirubakaran et al., 2019), making it a far simpler trait. Machine learning techniques like GRRF may be well‐suited to selecting loci associated with complex traits because all loci, and complex interactions between them are included in the adjustment of the model, allowing for the contribution of unassociated, but highly correlated loci (Barbosa et al., 2021). Simulated and empirical research has shown that incorporating machine learning methods (including random forest) into genome prediction and identifying relevant genomic regions of complex traits improves accuracy relative to linear mixed models and Bayesian approaches (Maldonado et al., 2020; Yin et al., 2020). Conversely, when examining genome prediction performance according to trait complexity as measured by number of qualitative trait loci, machine learning approaches (including random forest) outperformed Bayesian or linear mixed model techniques when relatively few qualitative trait loci (2–8) were simulated, but at increased trait complexities, all techniques were equally predictive. Other factors beyond trait complexity like heritance and dominance seem to play a significant role in determining the performance of machine learning approaches when identifying SNP loci (Alves et al., 2020; Barbosa et al., 2021).

A final reason for the difference in success of the methodologies could be due to the evolutionary timing of the divergence observed between traits. The evolution of the sex gene in gadoids occurred at least 45 million years ago (Kirubakaran et al., 2019), well before the divergence of spawning season groups in Atlantic cod from the wGOM. Most of the examples where the random forest technique is used to identify diagnostic loci are focused on traits that would have developed recently on an evolutionary time scale. For example, the GRRF method consistently outperformed the ranked *F*_ST_ method for assigning Atlantic cod to a population segment found within an MPA (Sinclair‐Waters, Bentzen, et al., 2018) and for discerning fine‐scale genetic differentiation in Atlantic salmon and Alaskan Chinook salmon populations (Sylvester et al., 2018). The random forest technique has also been used to successfully find diagnostic loci in Chinook salmon to separate seasonal reproductive run timing (Brieuc et al., 2015) and to distinguish salinity ecotypes in American eels (Pavey et al., 2015). While the exact mechanism that determines the efficiency of loci selection methodology is unknown, several techniques, including more traditional and machine learning approaches, should be utilized to maximize the chances of selecting a locus suite with the greatest assignment power.

The resulting SNP panels developed here will prove to be a valuable tool for fisheries research, management, and future stock assessments for Atlantic cod in the wGOM. With declining Atlantic cod biomass in the wGOM (NEFSC, 2013) and a severe decline in the recruitment of spring‐spawned Atlantic cod (Dean et al., 2019), there is a critical need to incorporate accurate tools that can be applied nonlethally into future stock assessments and research. The biology of Atlantic cod in the wGOM creates a complex and dynamic metapopulation structure, which should make the management of the fishery dynamic in turn. Incorporating all available information and tools into our understanding and management of wGOM Atlantic cod will provide the most accurate, concrete, and useful data to enact and evaluate any future regulatory changes.

## CONFLICT OF INTEREST

None declared.

## AUTHOR CONTRIBUTION

**Timothy O'Donnell:** Data curation (lead); Formal analysis (lead); Investigation (lead); Methodology (lead); Software (lead); Writing‐original draft (lead); Writing‐review & editing (lead). **Timothy James Sullivan:** Conceptualization (lead); Data curation (supporting); Formal analysis (supporting); Funding acquisition (lead); Methodology (supporting); Project administration (lead); Software (supporting); Supervision (lead); Writing‐review & editing (supporting).

## Data Availability

All data generated during the present study in the form of raw sequencing reads have been uploaded to NCBI’s Sequence Read Archive, accessible by bioproject number PRJNA669394, accession numbers SAMN16447555‐SAMN16447776. All scripts used during bioinformatic processing and Beagle format genotype likelihood data files for selected SNP panels were uploaded to Dryad, DOI accession number: https://doi.org/10.5061/dryad.ht76hdrg3.

## References

[ece37878-bib-0001] Alves, A. A. C. , da Costa, R. M. , Bresolin, T. , Fernandes, G. A. , Espigolan, R. , Ribeiro, A. M. F. , Carvalheiro, R. , & De Albuquerque, L. G. (2020). Genome‐wide prediction for complex traits under the presence of dominance effects in simulated populations using GBLUP and machine learning methods. Journal of Animal Science, 98(6), 1–11. 10.1093/JAS/SKAA179 PMC7367166 32474602

[ece37878-bib-0002] Anderson, E. C. (2010). Assessing the power of informative subsets of loci for population assignment: Standard methods are upwardly biased. Molecular Ecology Resources, 10(4), 701–710. 10.1111/j.1755-0998.2010.02846.x 21565075

[ece37878-bib-0003] Barbosa, I. D. P. , da Silva, M. J. , da Costa, W. G. , Castro Sant'Anna, I. , Nascimento, M. , & Cruz, C. D. (2021). Genome‐enabled prediction through machine learning methods considering different levels of trait complexity. Crop Science, 61(3), 1890–1902. 10.1002/csc2.20488

[ece37878-bib-0004] Barney, B. T. , Munkholm, C. , Walt, D. R. , & Palumbi, S. R. (2017). Highly localized divergence within supergenes in Atlantic cod (*Gadus morhua*) within the Gulf of Maine. BMC Genomics, 18(1), 1–14. 10.1186/s12864-017-3660-3 28359300 PMC5374575

[ece37878-bib-0005] Barth, J. M. I. , Berg, P. R. , Jonsson, P. R. , Bonanomi, S. , Corell, H. , Hemmer‐Hansen, J. , Jakobsen, K. S. , Johannesson, K. , Jorde, P. E. , Knutsen, H. , Moksnes, P. O. , Star, B. , Stenseth, N. C. , Svedäng, H. , Jentoft, S. , & André, C. (2017). Genome architecture enables local adaptation of Atlantic cod despite high connectivity. Molecular Ecology, 26(17), 4452–4466. 10.1111/mec.14207 28626905

[ece37878-bib-0006] Begg, G. A. , Friedland, K. D. , & Pearce, J. B. (1999). Stock identification and its role in stock assessment and fisheries management: An overview. Fisheries Research, 43(1–3), 1–8. 10.1016/S0165-7836(99)00062-4

[ece37878-bib-0007] Begg, G. A. , & Waldman, J. R. (1999). An holistic approach to fish stock identification. Fisheries Research, 43(1–3), 35–44. 10.1016/S0165-7836(99)00065-X

[ece37878-bib-0008] Berg, P. R. , Jentoft, S. , Star, B. , Ring, K. H. , Knutsen, H. , Lien, S. , Jakobsen, K. S. , & André, C. (2015). Adaptation to low salinity promotes genomic divergence in Atlantic Cod (*Gadus morhua L*.). Genome Biology and Evolution, 7(6), 1644–1663. 10.1093/gbe/evv093 25994933 PMC4494048

[ece37878-bib-0009] Berg, P. R. , Star, B. , Pampoulie, C. , Bradbury, I. R. , Bentzen, P. , Hutchings, J. A. , Jentoft, S. , & Jakobsen, K. S. (2017). Trans‐oceanic genomic divergence of Atlantic cod ecotypes is associated with large inversions. Heredity, 119(6), 418–428. 10.1038/hdy.2017.54 28930288 PMC5677996

[ece37878-bib-0010] Berg, P. R. , Star, B. , Pampoulie, C. , Sodeland, M. , Barth, J. M. I. , Knutsen, H. , Jakobsen, K. S. , & Jentoft, S. (2016). Three chromosomal rearrangements promote genomic divergence between migratory and stationary ecotypes of Atlantic cod. Scientific Reports, 6(1), 1–12. 10.1038/srep23246 26983361 PMC4794648

[ece37878-bib-0011] Bolger, A. M. , Lohse, M. , & Usadel, B. (2014). Trimmomatic: A flexible trimmer for Illumina sequence data. Bioinformatics, 30(15), 2114–2120. 10.1093/bioinformatics/btu170 24695404 PMC4103590

[ece37878-bib-0012] Bradbury, I. R. , Bowman, S. , Borza, T. , Snelgrove, P. V. R. , Hutchings, J. A. , Berg, P. R. , Rodríguez‐Ezpeleta, N. , Lighten, J. , Ruzzante, D. E. , Taggart, C. , & Bentzen, P. (2014). Long distance linkage disequilibrium and limited hybridization suggest cryptic speciation in Atlantic cod. PLoS One, 9(9), e106380. 10.1371/journal.pone.0106380 25259573 PMC4178228

[ece37878-bib-0013] Bradbury, I. R. , Hubert, S. , Higgins, B. , Borza, T. , Bowman, S. , Paterson, I. G. , Snelgrove, P. V. R. , Morris, C. J. , Gregory, R. S. , Hardie, D. C. , Hutchings, J. A. , Ruzzante, D. E. , Taggart, C. T. , & Bentzen, P. (2010). Parallel adaptive evolution of Atlantic cod on both sides of the Atlantic Ocean in response to temperature. Proceedings of the Royal Society B: Biological Sciences, 277(1701), 3725–3734. 10.1098/rspb.2010.0985 PMC2992707 20591865

[ece37878-bib-0014] Bradbury, I. R. , Hubert, S. , Higgins, B. , Bowman, S. , Borza, T. , Paterson, I. G. , Snelgrove, P. V. R. , Morris, C. J. , Gregory, R. S. , Hardie, D. , Hutchings, J. A. , Ruzzante, D. E. , Taggart, C. T. , & Bentzen, P. (2013). Genomic islands of divergence and their consequences for the resolution of spatial structure in an exploited marine fish. Evolutionary Applications, 6(3), 450–461. 10.1111/eva.12026 23745137 PMC3673473

[ece37878-bib-0015] Brieuc, M. S. O. , Ono, K. , Drinan, D. P. , & Naish, K. A. (2015). Integration of Random Forest with population‐based outlier analyses provides insight on the genomic basis and evolution of run timing in Chinook salmon (*Oncorhynchus tshawytscha*). Molecular Ecology, 24(11), 2729–2746. 10.1111/mec.13211 25913096

[ece37878-bib-0016] Broad Institute . (2019). Picard Toolkit. Retrieved from http://broadinstitute.github.io/picard/

[ece37878-bib-0017] Bureau, A. , Dupuis, J. , Hayward, B. , Falls, K. , & Van Eerdewegh, P. (2003). Mapping complex traits using Random Forests. BMC Genetics, 4(Suppl 1), 1–5. 10.1186/1471-2156-4-s1-s64 14975132 PMC1866502

[ece37878-bib-0018] Chen, K. Y. , Marschall, E. A. , Sovic, M. G. , Fries, A. C. , Gibbs, H. L. , & Ludsin, S. A. (2018). assignPOP: An r package for population assignment using genetic, non‐genetic, or integrated data in a machine‐learning framework. Methods in Ecology and Evolution, 9(2), 439–446. 10.1111/2041-210X.12897

[ece37878-bib-0019] Clucas, G. V. , Kerr, L. A. , Cadrin, S. X. , Zemeckis, D. R. , Sherwood, G. D. , Goethel, D. , Whitener, Z. , & Kovach, A. I. (2019). Adaptive genetic variation underlies biocomplexity of Atlantic Cod in the Gulf of Maine and on Georges Bank. PLoS One, 14(5), 1–25. 10.1371/journal.pone.0216992 PMC6534298 31125344

[ece37878-bib-0020] Clucas, G. V. , Lou, R. N. , Overgaard Therkildsen, N. , & Kovach, A. I. (2019). Novel signals of adaptive genetic variation in northwestern Atlantic cod revealed by whole‐genome sequencing. Evolutionary Applications, 12(10), 1971–1987. 10.1111/eva.12861 31700539 PMC6824067

[ece37878-bib-0021] COSEWIC . (2010). Assessment and Status Report on the Atlantic Cod (*Gadus morhua*) in Canada.

[ece37878-bib-0022] Dean, M. J. , Elzey, S. P. , Hoffman, W. S. , Buchan, N. C. , & Grabowski, J. H. (2019). The relative importance of sub‐populations to the Gulf of Maine stock of Atlantic cod. ICES Journal of Marine Science, 76(6), 1626–1640. 10.1093/icesjms/fsz083

[ece37878-bib-0023] Dean, M. J. , Hoffman, W. S. , Zemeckis, D. R. , & Armstrong, M. P. (2014). Fine‐scale diel and gender‐based patterns in behaviour of Atlantic cod (*Gadus morhua*) on a spawning ground in the Western Gulf of Maine. ICES Journal of Marine Science, 71(6), 1474–1489. 10.1093/icesjms/fsu040

[ece37878-bib-0024] Deng, H. (2013). Guided Random Forest in the RRF Package. arxiv:1306.0237

[ece37878-bib-0025] Deng, H. , & Runger, G. (2013). Gene selection with guided regularized random forest. Pattern Recognition, 46(12), 3483–3489. 10.1016/j.patcog.2013.05.018

[ece37878-bib-0026] Hemmer‐Hansen, J. , Nielsen, E. E. , Therkildsen, N. O. , Taylor, M. I. , Ogden, R. , Geffen, A. J. , Bekkevold, D. , Helyar, S. , Pampoulie, C. , Johansen, T. , & Carvalho, G. R. (2013). A genomic island linked to ecotype divergence in Atlantic cod. Molecular Ecology, 22(10), 2653–2667. 10.1111/mec.12284 23611647

[ece37878-bib-0027] Huret, M. , Runge, J. A. , Chen, C. , Cowles, G. , Xu, Q. , & Pringle, J. M. (2007). Dispersal modeling of fish early life stages: Sensitivity with application to Atlantic cod in the western Gulf of Maine. Marine Ecology Progress Series, 347, 261–274. 10.3354/meps06983

[ece37878-bib-0028] Jun, G. , Wing, M. K. , Abecasis, G. R. , & Kang, H. M. (2015). An efficient and scalable analysis framework for variant extraction and refinement from population‐scale DNA sequence data. Genome Research, 25(6), 918–925. 10.1101/gr.176552.114 25883319 PMC4448687

[ece37878-bib-0029] Karlsen, B. O. , Klingan, K. , Emblem, Å. , Jørgensen, T. E. , Jueterbock, A. , Furmanek, T. , Hoarau, G. , Johansen, S. D. , Nordeide, J. T. , & Moum, T. (2013). Genomic divergence between the migratory and stationary ecotypes of Atlantic cod. Molecular Ecology, 22(20), 5098–5111. 10.1111/mec.12454 23998762

[ece37878-bib-0030] Kerr, L. A. , Cadrin, S. X. , & Secor, D. H. (2010). Simulation modelling as a tool for examining the consequences of spatial structure and connectivity on local and regional population dynamics. ICES Journal of Marine Science, 67(8), 1631–1639. 10.1093/icesjms/fsq053

[ece37878-bib-0031] Kess, T. , Bentzen, P. , Lehnert, S. J. , Sylvester, E. V. A. , Lien, S. , Kent, M. P. , Corey, M. S. , Wringe, B. , Fairweather, R. , & Bradbury, I. R. (2019). Modular chromosome rearrangements reveal parallel and nonparallel adaptation in a marine fish. Ecology and Evolution, 10(2), 638–653. 10.1002/ece3.5828 PMC6988541 32015832

[ece37878-bib-0032] Kirubakaran, T. G. , Andersen, Ø. , De Rosa, M. C. , Andersstuen, T. , Hallan, K. , Kent, M. P. , & Lien, S. (2019). Characterization of a male specific region containing a candidate sex determining gene in Atlantic cod. Scientific Reports, 9(1), 1–9. 10.1038/s41598-018-36748-8 30644412 PMC6333804

[ece37878-bib-0033] Kirubakaran, T. G. , Grove, H. , Kent, M. P. , Sandve, S. R. , Baranski, M. , Nome, T. , De Rosa, M. C. , Righino, B. , Johansen, T. , Otterå, H. , Sonesson, A. , Lien, S. , & Andersen, Ø. (2016). Two adjacent inversions maintain genomic differentiation between migratory and stationary ecotypes of Atlantic cod. Molecular Ecology, 25(10), 2130–2143. 10.1111/mec.13592 26923504

[ece37878-bib-0034] Korneliussen, T. S. , Albrechtsen, A. , & Nielsen, R. (2014). ANGSD: Analysis of Next Generation Sequencing Data. BMC Bioinformatics, 15(1), 1–14. 10.1186/s12859-014-0356-4 25420514 PMC4248462

[ece37878-bib-0035] Kovach, A. I. , Breton, T. S. , Berlinsky, D. L. , Maceda, L. , & Wirgin, I. (2010). Fine‐scale spatial and temporal genetic structure of Atlantic cod off the Atlantic coast of the USA. Marine Ecology Progress Series, 410, 177–195. 10.3354/meps08612

[ece37878-bib-0036] Langmead, B. , & Salzberg, S. L. (2012). Fast gapped‐read alignment with Bowtie 2. Nature Methods, 9(4), 357–359. 10.1038/nmeth.1923 22388286 PMC3322381

[ece37878-bib-0037] Li, H. , Handsaker, B. , Wysoker, A. , Fennell, T. , Ruan, J. , Homer, N. , Marth, G. , Abecasis, G. , & Durbin, R. (2009). The Sequence Alignment/Map format and SAMtools. Bioinformatics, 25(16), 2078–2079. 10.1093/bioinformatics/btp352 19505943 PMC2723002

[ece37878-bib-0038] Maldonado, C. , Mora‐Poblete, F. , Contreras‐Soto, R. I. , Ahmar, S. , Chen, J. T. , do Amaral Júnior, A. T. , & Scapim, C. A. . (2020). Genome‐wide prediction of complex traits in two outcrossing plant species through deep learning and Bayesian regularized neural network. Frontiers in Plant Science, 11(November), 1–14. 10.3389/fpls.2020.593897 33329658 PMC7728740

[ece37878-bib-0039] McBride, R. S. , Ames, T. , Andrushchenko, I. , Cadrin, S. X. , Cournane, J. M. , Dean, M. , Zemeckis, D. R. (2021). Atlantic cod stock structure working group draft proposed biological stocks.

[ece37878-bib-0040] McKenna, A. , Hanna, M. , Banks, E. , Sivachenko, A. , Cibulskis, K. , Kernytsky, A. , Garimella, K. , Altshuler, D. , Gabriel, S. , Daly, M. , & DePristo, M. A. (2010). The genome analysis Toolkit: A MapReduce framework for analyzing next‐generation DNA sequencing data. Genome Research, 20(9), 1297–1303. 10.1101/gr.107524.110 20644199 PMC2928508

[ece37878-bib-0041] Meisner, J. , & Albrechtsen, A. (2018). Inferring population structure and admixture proportions in low‐depth NGS data. Genetics, 210(2), 719–731. 10.1534/genetics.118.301336 30131346 PMC6216594

[ece37878-bib-0042] Northeast Fisheries Science Center . (2013). Northeast Regional Stock Assessment Workshop (55th SAW). Assessment Summary Report. *Northeast Fisheries Science Center*, *January*, 42. Retrieved from http://www.asmfc.org/uploads/file/crd1218.pdf

[ece37878-bib-0043] Northeast Fisheries Science Center . (2021). Analyzing Cod Populations in the Atlantic. Retrieved from https://www.fisheries.noaa.gov/new‐england‐mid‐atlantic/commercial‐fishing/analyzing‐cod‐populations‐atlantic

[ece37878-bib-0044] Pavey, S. A. , Gaudin, J. , Normandeau, E. , Dionne, M. , Castonguay, M. , Audet, C. , & Bernatchez, L. (2015). RAD Sequencing Highlights Polygenic Discrimination of Habitat Ecotypes in the Panmictic American Eel. Current Biology, 25(12), 1666–1671. 10.1016/j.cub.2015.04.062 26028437

[ece37878-bib-0045] Pershing, A. J. , Alexander, M. A. , Hernandez, C. M. , Kerr, L. A. , Le Bris, A. , Mills, K. E. , Nye, J. A. , Record, N. R. , Scannell, H. A. , Scott, J. D. , Sherwood, G. D. , & Thomas, A. C. (2015). Slow adaptation in the face of rapid warming leads to collapse of the Gulf of Maine cod fishery. Science, 350(6262), 809–812. 10.1126/science.aac9819 26516197

[ece37878-bib-0046] R Core Team (2020). R: A Language and Environment for Statistical Computing. R Foundation for Statistical Computing. https://www.r‐project.org/

[ece37878-bib-0047] Rodríguez‐Ramilo, S. T. , Baranski, M. , Moghadam, H. , Grove, H. , Lien, S. , Goddard, M. E. , Meuwissen, T. H. E. , & Sonesson, A. K. (2019). Strong selection pressures maintain divergence on genomic islands in Atlantic cod (*Gadus morhua L*.) populations. Genetics Selection Evolution, 51(1), 1–15. 10.1186/s12711-019-0503-5 PMC6819574 31664896

[ece37878-bib-0048] Sinclair‐Waters, M. , Bentzen, P. , Morris, C. J. , Ruzzante, D. E. , Kent, M. P. , Lien, S. , & Bradbury, I. R. (2018). Genomic tools for management and conservation of Atlantic cod in a coastal marine protected area. Canadian Journal of Fisheries and Aquatic Sciences, 75(11), 1915–1925. 10.1139/cjfas-2017-0254

[ece37878-bib-0049] Sinclair‐Waters, M. , Bradbury, I. R. , Morris, C. J. , Lien, S. , Kent, M. P. , & Bentzen, P. (2018). Ancient chromosomal rearrangement associated with local adaptation of a postglacially colonized population of Atlantic Cod in the northwest Atlantic. Molecular Ecology, 27(2), 339–351. 10.1111/mec.14442 29193392

[ece37878-bib-0050] Sodeland, M. , Jorde, P. E. , Lien, S. , Jentoft, S. , Berg, P. R. , Grove, H. , Kent, M. P. , Arnyasi, M. , Olsen, E. M. , & Knutsen, H. (2016). “Islands of Divergence” in the Atlantic cod genome represent polymorphic chromosomal rearrangements. Genome Biology and Evolution, 8(4), 1012–1022. 10.1093/gbe/evw057 26983822 PMC4860689

[ece37878-bib-0051] Star, B. , Tørresen, O. K. , Nederbragt, A. J. , Jakobsen, K. S. , Pampoulie, C. , & Jentoft, S. (2016). Genomic characterization of the Atlantic cod sex‐locus. Scientific Reports, 6, 1–9. 10.1038/srep31235 27499266 PMC4976360

[ece37878-bib-0052] Sylvester, E. V. A. , Bentzen, P. , Bradbury, I. R. , Clément, M. , Pearce, J. , Horne, J. , & Beiko, R. G. (2018). Applications of random forest feature selection for fine‐scale genetic population assignment. Evolutionary Applications, 11(2), 153–165. 10.1111/eva.12524 29387152 PMC5775496

[ece37878-bib-0053] Therkildsen, N. O. , Hemmer‐Hansen, J. , Hedeholm, R. B. , Wisz, M. S. , Pampoulie, C. , Meldrup, D. , Bonanomi, S. , Retzel, A. , Olsen, S. M. , & Nielsen, E. E. (2013). Spatiotemporal SNP analysis reveals pronounced biocomplexity at the northern range margin of Atlantic cod *Gadus morhua* . Evolutionary Applications, 6(4), 690–705. 10.1111/eva.12055 23789034 PMC3684748

[ece37878-bib-0054] Therkildsen, N. O. , & Palumbi, S. R. (2017). Practical low‐coverage genomewide sequencing of hundreds of individually barcoded samples for population and evolutionary genomics in nonmodel species. Molecular Ecology Resources, 17(2), 194–208. 10.1111/1755-0998.12593 27496322

[ece37878-bib-0055] Tørresen, O. K. , Star, B. , Jentoft, S. , Reinar, W. B. , Grove, H. , Miller, J. R. , Walenz, B. P. , Knight, J. , Ekholm, J. M. , Peluso, P. , Edvardsen, R. B. , Tooming‐Klunderud, A. , Skage, M. , Lien, S. , Jakobsen, K. S. , & Nederbragt, A. J. (2017). An improved genome assembly uncovers prolific tandem repeats in Atlantic cod. BMC Genomics, 18(1), 1–23. 10.1186/s12864-016-3448-x 28100185 PMC5241972

[ece37878-bib-0056] Vu, V. Q. (2011). ggbiplot: A ggplot2 based biplot. Retrieved from http://github.com/vqv/ggbiplot

[ece37878-bib-0057] Wickham, H. (2016). ggplot2: Elegant Graphics for Data Analysis. Springer‐Verlag. https://ggplot2.tidyverse.org

[ece37878-bib-0058] Yin, L. , Zhang, H. , Zhou, X. , Yuan, X. , Zhao, S. , Li, X. , & Liu, X. (2020). KAML: Improving genomic prediction accuracy of complex traits using machine learning determined parameters. Genome Biology, 21(1), 1–22. 10.1186/s13059-020-02052-w PMC7386246 32552725

[ece37878-bib-0059] Zemeckis, D. R. , Hoffman, W. S. , Dean, M. J. , Armstrong, M. P. , & Cadrin, S. X. (2014). Spawning site fidelity by Atlantic cod (*Gadus morhua*) in the Gulf of Maine: Implications for population structure and rebuilding. ICES Journal of Marine Science, 71(6), 1356–1365. 10.1093/icesjms/fsu117

[ece37878-bib-0060] Zemeckis, D. R. , Martins, D. , Kerr, L. A. , & Cadrin, S. X. (2014). Stock identification of Atlantic cod (*Gadus morhua*) in US waters: An interdisciplinary approach. ICES Journal of Marine Science, 71(6), 1490–1506. 10.1093/icesjms/fsu032

